# Schizophrenic Patients between General and Forensic Psychiatry

**DOI:** 10.3389/fpubh.2016.00135

**Published:** 2016-06-30

**Authors:** Fanny de Tribolet-Hardy, Elmar Habermeyer

**Affiliations:** ^1^Department for Forensic Psychiatry, University Hospital of Psychiatry Zurich, Zurich, Switzerland

**Keywords:** forensic psychiatry, schizophrenia, patients, violent offences, general psychiatry

Because of exploding costs and the need for new (high-secure) facilities, forensic measures have become major points of discussion within politics, within economics, and within social levels. The increase of beds in forensic psychiatry has been reported within several European countries, Canada, and the United States (1, 2). Approximately 50–60% of the patients within forensic measure suffer from schizophrenic disorders [e.g., Ref. (1)]. This may not be surprising due to the well-known association between schizophrenia and higher risk for violent offenses (3–5).

Despite the consistently found association between schizophrenia and violent offending the relevance of the disease itself for offending remains somewhat unclear and is discussed controversially. Most prominently, comorbid disorders, especially substance dependence or abuse and antisocial personality disorder, non-compliance toward medication and/or treatment, suicidal behavior, or previous violent offenses are known to be risk factors for later (violent) offending (4, 6). Together, these factors form a high-risk cluster of features of schizophrenic patients in terms of future violent behavior but do also rather reflect the multifactorial genesis of criminal acts, than actually explaining the increasing numbers within forensic psychiatry.

Consequently, other explanations focus on legal, societal, or organizational problems. For example, Schanda et al. (7) pointed out legal issues due to a softening of the criteria “outstanding dangerousness” for being pledged as not guilty by reason of insanity within Austrian law. Between 1990 and 2007 judgments of not guilty by reason of insanity because of minor or “disturbing” offenses, as threatening, compulsion, or obstructing an officer, rose from 20 to 50% in Austria. Similarly, other authors conclude that the increase of forensic patients is not due to higher crime rates or the increase of mental illness but rather because of the increased focus on security and public protection (1). These political and social developments, however, lead to higher risk for severe mentally ill people to get in contact with criminal justice system because of minor disruptions. Crocker and Côté (8) described a tendency that criminal justice has become a “point of entry” for mental health care.

Another frequently discussed point is the shift within general psychiatry during the past decades. As every other health-care provider general psychiatry entered the corporate sector, which put optimization, profit and collective negotiations with health insurances on the agenda (1, 9, 10). Anyhow, it has been shown that possible negative consequences of this development (e.g., short duration of inpatient or/and outpatient treatment, high rates of previous psychiatric admissions) are not associated with violence of schizophrenic patients (11). Furthermore, Priebe et al. (2) state that the increase of forensic beds is not associated with the decrease of conventional psychiatric beds. In fact, patients often leave hospital against medical advice or escape (12). Consequently, it is likely that high drop-out rates in general psychiatry rather than the decrease of beds in general psychiatry is responsible for assumed transfer of patients from general psychiatry to forensic psychiatry. Especially, patients with the preliminary described high-risk features show withdrawal after dismissal due to antisocial traits or/and comorbid substance abuse (13).

The obvious problem that a distinct group of problematic schizophrenic patients is slipping through the fingers of general psychiatric care has been shown in several European cohorts. With percentages between 75 and 85, most schizophrenic patients in forensic measure have been repeatedly admitted into psychiatric hospitals before committing their index offense (9, 10, 14). Between first hospitalization and index offense, a timeframe of 6.8 years with on average five to six hospitalizations is reported (10, 14). In a German cohort, most of the patients have been known as “problematic” within general psychiatry and during outpatient care before their index offense which resulted in lower contact rates (14). Other findings prompt that the risk factor for previous interpersonal violence or damage of property is seldom (13 vs. 2%) inquired by psychiatric staff (15). Hence, early detection of a high-risk group of schizophrenic patients has been emphasized frequently. Nevertheless, rising numbers within forensic measures suggest that either detection of high-risk patients or necessary treatment is not provided sufficiently (12).

Another yet not verified shift within general psychiatry, which may be associated with the increasing numbers of forensic patients, is the growing reluctance toward coercive measures. As shown in different studies, especially schizophrenic patients with acute symptoms are associated with higher rates of coercive measures (16). But, low numbers in coercive measures have become some sort of seal of quality for psychiatric hospitals (17, 18). A total reluctance toward coercive measures in general psychiatry may lead toward an outsourcing of “difficult” patients into forensic psychiatry, where public understanding for coercive measures may be greater due to the criminal background of the patient.

Taking together, there seems to be a high-risk group of severely and chronically ill schizophrenic patients who is currently not or cannot be detected and not treated sufficiently within general psychiatry. Legal regulations as well as social and political developments toward a no risk policy in terms of public protection lowered the barriers for mentally ill people to get in contact with criminal justice. Because of several admissions into psychiatric hospitals before offending, a timeframe of about 7 years between first admission and index offense at least in German populations, preventive measures should be initiated in general psychiatry. Important steps should include (a) a screening of risk factors for violence, (b) secure and specialized inpatient cares that (c) allow longer in-stays and (d) provide treatment options concerning antisocial behavior, comorbid substance, and medical compliance. Finally, (e) solid community supervision has to be organized before the patient is discharged.

## Author Contributions

FT-H drafted the first version of the manuscript. EH gave a substantial contribution to the drafting and critically revised the article for important intellectual content. Both authors approved the final version for publication.

## Conflict of Interest Statement

The authors declare that the research was conducted in the absence of any commercial or financial relationships that could be construed as a potential conflict of interest.
